# A Comparative Study of the Sodium Content and Calories from Sugar in Toddler Foods Sold in Low- and High-Income New York City Supermarkets

**DOI:** 10.5539/gjhs.v6n5p22

**Published:** 2014-05-07

**Authors:** Lalitha Samuel, Danna Ethan, Corey Basch, Benny Samuel

**Affiliations:** 1 Dietetics, Foods, and Nutrition, Department of Health Sciences, Lehman College, The City University of New York, Bronx, NY, USA; 2 Health Education and Promotion, Department of Health Sciences, Lehman College, The City University of New York, Bronx, NY, USA; 3 Department of Public Health, William Paterson University, Wayne, NJ, USA; 4 Brookdale University Hospital, Brooklyn, NY, USA

**Keywords:** toddler foods, high-sodium, supermarkets, high-income, low-income

## Abstract

Information from the nutrition facts labels of toddler foods marketed in low- and high-income New York City zip codes were analyzed for sodium content, the proportion of sugar-derived calories, and presence of sugar and/or high-fructose corn syrup as an added sweetener in the list of ingredients. Among the 272 toddler foods analyzed, more than a quarter were high in sodium, over one-third derived at least 20% their calories from sugar, and more than 41% of the foods had sugar and/or high-fructose corn syrup listed among the first five ingredients. The proportion of foods with such nutritional characteristics did not significantly differ between the low- and high-income neighborhood supermarkets. Median sodium content was highest among “side dishes” and “meals.” The proportion of calories derived from sugar was found to be highest among “snacks and yogurt blends” in both low- and high-income neighborhoods and “breakfast foods and cereals” in low-income neighborhoods. When compared to high-income neighborhoods, more than three times the proportion of total calories in “breakfast foods and cereals” sold in low-income neighborhoods were derived from sugar. Since taste preferences established during childhood can have long-lasting influence on dietary habits, it is imperative to limit the promotion of toddler foods that are high in sodium and sugar as well as educate parents to make nutritionally sound decisions at the point of purchase.

## 1. Introduction

Toddlerhood is a vital stage in childhood development. As children transition from an all-milk diet in infancy to family food, this stage is pivotal in establishing healthy eating habits. Children develop food preferences at home, and dietary habits developed during childhood last into adulthood (Gruber & Haldeman, 2009). Early exposure during childhood to foods high in sugar and salt has been related to their increased consumption and preference for these foods and increased risk for chronic diseases during adulthood (Hill, 2002; Elliott & Conlon, 2010; Elliott, 2010). Research directed towards toddler nutrition has focused on nutrient intake and portion sizes (Briefel, Reidy, Karwe, Jankowski, & Hendricks, 2004; More, 2013; Chaidez, McNiven, Vosti, & Kaiser, 2013). Very few studies have analyzed the nutritional quality of toddler foods sold in supermarkets and grocery stores (Elliott & Conlon, 2010; Elliott, 2010) and we identified no studies that compare the nutritional quality of these food products based on placement in high- versus low-income areas. Research has established that limited access to nutritious foods impacts health (Policy Link & Food Trust, 2010; Robert Wood Johnson Foundation, 2008). Studies have compared the cost and quality of food items promoted in high- and low-income zip codes. This comparison has occurred across different contexts, including promotions at fast food restaurants (Basch, Ethan, & Rajan, 2013), and grocery store circulars (Ethan, Basch, Rajan, & Samuel, 2013). In general, less nutrient-dense foods are most heavily promoted and advertised in low-income neighborhoods (Grier & Kumanyika, 2008; Yancey et al., 2009; Cummins & Macintyre, 2006).

In light of the knowledge gap regarding the nutritional quality of toddler foods, this study analyzed the proportion of 1) sugar-derived calories in toddler foods, 2) toddler foods that had sugar and/or high fructose syrup listed among the first five ingredients and, 3) toddler foods that were high in sodium content. We also analyzed whether the proportion of toddler foods with these nutritional characteristics varied between the highest- and lowest-income areas of New York City.

## 2. Methods

### 2.1 Sampling Frame

Using current United States Census data (USCB, 2013), median household income was used to identify five highest- and lowest-income zip codes in New York City. Supermarket chains were identified and verified through telephone numbers and addresses accessed from three online databases (Yahoo local, White Pages and Yellow Pages). When a supermarket was absent in a given zip code, stores in the zip code with the next lowest (or highest) median income were identified. When two or more franchises of the same store were present in the same zip code, one was randomly selected for the study.

A preliminary visit to two local supermarkets outside the sampling frame was done to identify the manufacturers of toddler foods. A detailed coding sheet was then developed that included the names, description and nutritional information for toddler foods advertised on the website of each manufacturer identified during the preliminary visits. Toddler foods were identified as snacks, meals, breakfast foods, side dishes and yogurt blends packaged in tubs, bowls, pouches, cartons and jars with the label or website identifying them as “toddler foods”, “12 months onwards,” “12 months +,” “toddler 2+,” or “stage 4”. All simple fruit and/or vegetable purees were excluded as these generally do not contain added sugar or salt (Elliott, 2010). The coding sheet was pilot-tested by two researchers who independently identified toddler foods in three stores (outside the sampling frame) with an inter-rater agreement of over 91%. This coding sheet allowed for systematic and consistent recording of data from stores within the sampling frame.

For the purpose of data collection, researchers visited the stores within the sampling frame and used the coding sheet to identify toddler foods sold in each store. For toddler foods present in a store but absent on the coding sheet, the name and full description of the product, manufacturer’s name as well as nutritional information was recorded on site from the nutrient facts label of the product. After obtaining the names and description of all toddler foods on site, they were categorized as either: 1) breakfast foods and cereals, 2) meals, 3) side dishes or, 3) snacks and yogurt blends, based on their identification on the manufacturer’s website.

### 2.2 Nutritional Data

Information regarding calories, sodium and sugar content per serving was obtained from the nutrition facts label and recorded on the coding sheet. The list of ingredients was examined to identify the presence of sugar and/or high fructose corn syrup among the first five ingredients. Per guidelines outlined by the Food and Drug Administration (FDA), foods were classified as “high” in sodium content if a serving of food contains “20% or above” the daily value (DV) for sodium (FDA, 2012). Thus, using a DV of 1500 milligrams per day recommended by the Institute of Medicine (IOM) for 1-3 year old children (IOM, 2005), toddler foods containing sodium amounts of at least 300 milligrams per serving (mg/s), were classified as “high-sodium.” In the case of sugar content, toddler foods were considered to be high in sugar if 20% or more of their calories were derived from sugars. This approach, previously used in a content analysis study of baby foods sold in Canadian stores (Elliott, 2010), uses total sugar content quantified on the product’s nutrition facts label to calculate the sugar-derived calories as a percentage of the total calories in one serving of the food.

### 2.3 Data Analysis

All collected data were cleaned and warehoused onto a spreadsheet. The total number of products that qualified as “breakfast foods and cereals,” “meals,” “side dishes,” and “snacks and yogurt blends” in the high- and low-income zip codes was calculated. The total number of high- and low-sodium foods, foods that had sugar and/or high fructose corn syrup listed among the first five ingredients, and foods that had more than 20% of their calories from sugar in both high- and low-income zip codes were also calculated. Statistical tests were used to identify if the proportion of marketed toddler foods with aforementioned characteristics were significantly different between the two zip code groups. All data calculation and analyses were done on Microsoft Excel (Version 12).

## 3. Results

A total of 29 stores: 12 in the highest-income and 17 in the lowest-income zip codes of New York City were identified and verified for the study. Within this sampling frame, a total of 272 toddler foods were analyzed, of which 43% (n=116) were from low-income zip codes and 57% (n=156) were from high-income zip codes. An independent t-test indicated no significant difference in the mean number of toddler foods sold by supermarket chains in the low- and high-income neighborhoods (p>0.05).

Table 1 shows the distribution of different food categories as well as the proportion of high- and low-sodium toddler foods identified in this study. Meal products represented almost half (48%, n=131) of the toddler foods in the sample. “Side dishes” (5%, n=13) and “breakfast foods and cereals” (10%, n=26) had the lowest representation while “snacks and yogurt blends” represented a little more than a third of the foods (38%, n=102). The proportions of these food categories did not differ significantly between the low- and high-income neighborhoods, *X_2_*(3, N=272)=0.179, *p*>0.05. In terms of nutritional quality, more than a quarter of the foods were high in sodium (27%, n=74), 41% had at least 20% of their calories derived from sugar (n=112) and a little more than a third had sugar and/or high fructose corn syrup listed among the first five ingredients (37%, n=101). The proportion of foods with these nutritional attributes was not significantly different between the low- and high-income neighborhoods, *X_2_*(2, N=272)=0.178, *p*>0.05.

**Table 1:** Proportion of Different Toddler Food Categories, High-sodium Toddler Foods, and Toddler Foods with at least 25% Sugar-Derived Calories Sold in Low- and High-income New York City Zip Codes

Product description of toddler foods	Percent proportion of different toddler foods		Total
	Low-income zip codes	High-income zip codes	
Total number of toddler foods	42.6%	57.4%	272
Breakfast foods	7.8%	10.9%	26
Meals	51.7%	45.5%	131
Side dishes	6.0%	3.8%	13
Snacks and yogurt blends	34.5%	39.7%	102
High-sodium foods	27.6%	26.9%	74
High-sodium breakfast foods	0	0	0
High-sodium meals	43.3%	42.3	56
High-sodium side dishes	85.7%	100	12
High-sodium snacks and yogurt blends	0%	9.7%	6
Foods with at least 20% of their calories from sugar	37.1%	44.2%	112
Foods with sugar, sucrose and/or high fructose corn syrup listed among the first five ingredients	50.9%	26.9%	101
Foods with at least 20% of sugar-derived calories that have sugar, sucrose and/or high fructose corn syrup listed among the first five ingredients	60.5%	26.1%	44
Breakfast foods and cereals with sugar, sucrose and/or high fructose corn syrup listed among the first five ingredients	55.6%	23.5%	9
Meals with sugar, sucrose and/or high fructose corn syrup listed among the first five ingredients	46.7%	33.8%	52
Side dishes with sugar, sucrose and/or high fructose corn syrup listed among the first five ingredients	85.7%	50.0%	69
Snacks and yogurt blends with sugar, sucrose and/or high fructose corn syrup listed among the first five ingredients	50.0%	17.7%	31

### 3.1 Sodium Content

A little more than a quarter of the toddler foods sold in both low-income (28%, n=32) and high-income (27%, n=42) neighborhoods were high in sodium (Table 1). The greatest proportions of high-sodium foods were in the categories of side dishes (92%, n=12). The median sodium content in this food group was equivalent to the threshold of 300 mg/s used for the categorization of high-sodium toddler foods (Table 2). Although more than 42% of toddler meals were high-sodium (n=56), the median sodium content in this food group was less than the 300 mg/s threshold used for categorization of high-sodium foods. An independent t-test confirmed no significant difference in the representation of high-sodium foods in these two food categories sold in low- and high-income neighborhoods (p>0.05). Although high-sodium foods were absent or present in very low proportion in the “breakfast foods and cereals” and “snacks and yogurt blends” categories, sodium content was found to be significantly higher in these two products categories sold in high-income neighborhoods (p<0.05).

**Table 2:** Sodium Content and Calorie Contribution from Sugars in Toddler Foods Sold in Low- and High-income New York City Zip Codes

Toddler food category	Median sodium content*^a^* (interquartile range)		Median calorie contribution*^b^* from sugars (interquartile range)d	

	Low-income zip codes	High-income zip codes	Low-income zip codes	High-income zip codes
Breakfast foods	130.0 (10.0-130.0)	130.0 (120.0-150.0)	31.4 (31.4-36.9)	10.0 (10.0-22.5)
Meals	240.0 (190.0-330.0)	280.0 (180.0-330.0)	10.0 (5.7-12.5)	8.0 (3.6-12.0)
Side dishes	300.0 (300.0-300.0)	300.0 (300.0-300.0)	3.6 (3.6-3.6)	3.6 (3.6-3.6)
Snacks and yogurt blends	25.0 (20.0-51.3)	45.0 (20-92.5)	34.3 (13.3-54.0)	26.7 (25.0-45.7)

a milligram per serving (mg/s);

b per cent (%)

### 3.2 Presence of Added Sweeteners and Proportion of Sugar-Derived Calories

A little more than a third of the products (37%, n=101) had sugar and/or high fructose corn syrup listed among the first five ingredients (Table 1). The proportion of such foods was almost twice in the low-income neighborhood stores (51%, n=59) as compared to the high-income stores (27%, n=42). The proportion of foods with these added sweeteners listed among the first five ingredients was highest among side dishes (69%, n=9). As compared to their high-income counterparts, more than twice the proportion of breakfast foods and cereals (24% versus 56%) and almost three times the proportion of snacks and yogurt blends (18% versus 50%) sold in low-income neighborhoods listed added sweeteners among the first five ingredients (Table 1).

While the proportion of toddler foods that derived 20% or more of their calories from sugar (per serving) was slightly higher in the high-income neighborhoods, more than 60% of such products sold in the low-income neighborhoods had sucrose and/or high fructose corn syrup among the first five ingredients. In contrast, a little over a quarter (26%) of such products sold in high-income neighborhoods had these sweeteners among the first five ingredients (Table 1). The median calorie contribution from sugars (per serving) in breakfast foods and cereals sold in the low-income neighborhoods (31%) was more than thrice that of their counterparts from high-income neighborhoods (10%) (Table 2). Although the proportion of sugar-derived calories was higher for all the food categories sold in low-income neighborhoods, an independent t-test showed that the difference was significant only for breakfast foods and cereals (p<0.05).

## 4. Discussion

Population-based strategies for reducing the prevalence of hypertension, cardiovascular disease, metabolic syndrome and diabetes have focused in part on the reduction of sodium and sugar added to food products. These measures are predominantly devoted to adults. Current research suggests that dietary habits early on in life can alter gene expression to impact body composition during adulthood (Maurer, Chen, McPherson & Reimer, 2009). Nutrition during early childhood and toddlerhood can have a long-lasting influence on an individual’s health as dietary habits and taste preferences developed in this stage can pave the way for adopting a healthy diet during adulthood (Reverdy, Schlich, Köster, Ginon & Lange, 2010; Infant & Toddler Forum, 2009). A recent study found that more than 60% of the baby foods analyzed from Canadian stores were high in sodium or had a high proportion of sugar-derived calories (Elliott, 2010). Another study reported on the high sodium content of commercial infant and toddler foods sold in grocery stores in the United States (Maalouf, Cogswell, Gunn & Merritt, 2013). Against this background, this study assessed these nutritional characteristics of toddler foods sold in low- and high-income neighborhood supermarkets of New York City and whether these qualities differed between these neighborhoods.

Our findings concluded no significant difference in the proportion of toddler foods that were high in sodium, had sugar and/or high fructose corn syrup listed among the first five ingredients, as well as foods with more than 20% of sugar-derived calories marketed in low- and high-income neighborhoods. Our findings that more than a quarter of the toddler foods sold in both neighborhoods were high-sodium indicate the need for cohesive measures by the food industry to reduce sodium levels in toddler foods. Salt, which comprises 40% sodium is added to processed foods as a flavor enhancer and appetite stimulator (Lesham, 2009) as well as to inhibit microbial food spoilage (Durack, Alonso-Gomez & Wilkinson, 2013).

More than 41% of the analyzed foods had at least 20% of their calories from sugar, the median contribution being highest for “snacks and yogurt blends” in both low- and high-income neighborhoods and “breakfast foods and cereals” sold in low-income neighborhoods. Consumption of sugar-sweetened breakfast cereals has been identified as a significant contributor to added sugar intake among Americans (Harvard School of Public health [HSPH], 2014). In children, sweetened breakfast cereals have been linked to larger portion sizes and reduced nutritional quality (Harris, Schwartz, Ustjanauskas, Ochri-Vachaspati, & Brownell, 2010) and contribute up to 9% of the added sugars in children’s diet (Guthrie & Morton, 2000). It has been shown that not only are taste preferences for sweetness established in the early years of life (Birch, 1999), but that sweetness is one of the sensory parameters that drive food preferences during childhood (Wardle, Sanderson, Gibson, & Rapoport, 2002). In addition to the high palatability of processed foods with added sugars and fats, the low cost attributed to these foods has been a contributing factor to the obesogenic effect of such foods (Drewnowski &Specter, 2002), especially in children (Harrison & Marske, 2005) and in low-income neighborhoods (Grier & Kumanyika, 2008; Yancey et al., 2009). Previous research has indicated that palatability of toddler foods in not significantly altered by omitting added sugars and avoidance of added sugars can thus be used to limit the energy density of these foods (Bouhlal et al., 2011). Our findings that low-income neighborhoods had a greater proportion of toddler foods with added sugars as well as a higher median value for the percent calorie contribution from sugars confirm previous findings that foods with added sugars are more heavily promoted in low-income neighborhoods (Grier & Kumanyika. 2008; Yancey et al., 2009; Cummins & Macintyre, 2006).

This study was limited in its cross-sectional nature and therefore did not address food-purchasing trends, i.e. certain brands may have a higher turnover and newer brands and/or flavors may be added periodically. Second, the sugar content on the nutrition facts label was used to derive the percentage contributing to calories in the food. This nuanced approach, though unable to differentiate between sugars naturally present in foods and those added during processing, has been used previously in analyzing the nutritional quality of infant foods (Elliott, 2010). Our analysis of the food ingredients label for the presence of sugar and/or high fructose corn syrup among the first five ingredients provided an added objective measure for the presence of significant amount of added sweeteners, although the proportion of calories from these added sugars could not be determined. Third, in order keep this analysis objective and consistent, the study limited its identification of added sugars to the presence of sugar and/or high fructose corn syrup among the first five components in the ingredients list. Added sweeteners such as cane syrup, sugarcane juice and brown sugar are frequently added to foods and, are metabolized just like sugar thereby contributing to calories (HSPH, 2014; Academy of Nutrition and Dietetics, 2014; USDA, 2014). Thus, it is possible that our findings regarding the prevalence of toddler foods with added sweeteners are underestimated. Further, this study was limited to supermarket chains therefore does not completely represent the sale of packaged toddler foods in convenience stores and bodegas in the selected neighborhoods.

The findings from this study contribute to the dearth of literature on the nutritional quality of toddler food products available in supermarkets. In addition, we are aware of no other studies that assessed possible differences in nutritional quality of toddler foods based on placement in high- versus low-income area supermarkets. Although the proportion of high-sodium foods sold in high- and low-income neighborhoods were not significantly different, it is nonetheless important to note that, for both groups, more than a quarter of the toddler foods for sale were high in sodium. Further, over a third of the products had added sweeteners and more than 41% derived at least 20% of their calories derived from sugar. Salt concentrations have been shown to have positive correlation to portion sizes for children (Bouhlal, Issanchou & Nicklaus, 2011). A preference for salty foods developed during childhood can predispose them perceiving unsalted foods as being flavorless (The Food Commission, 2000), as well as possibly increase the risk for diseases in adulthood (Elliott & Conlon, 2011; Elliott, 2010). Free sugars have also been implicated in the development of dental diseases, which have been identified as non-communicable diseases with the highest global prevalence (WHO, 2014a). In the absence of recommended daily values for sugar intake, dietary guidelines therefore recommend minimizing the intake of foods with high amounts of sugars (USDA & USHHS, 2010). The World Health Organization (WHO) recommends that sugars should make up less than 10% of total energy intake per day and that consumption of sugars in amounts less than 5% of calorie intake would have additional benefits (WHO, 2014b). Complementing this research, WHO recommendations on the marketing of foods to children focus on reducing the impact of promoting foods that are high in sugars and salt (WHO, 2009).

These findings have important public health implications when considering this parent population’s likelihood of reading and understanding food labels and related impact on purchasing these food products. Food labels, when correctly interpreted, can empower parents to make nutritionally wise decisions when purchasing toddler foods. The United States Food and Drug Administration’s labeling regulations for foods aimed toward small children differ from those for foods targeted at older youth and adults (FDA, 2013). Specifically, the percent daily value is stated for protein, vitamins and minerals only. This suggests that parents have access to less information to make informed purchasing decisions and adequately compare products based on nutritional value. Parents should therefore be encouraged to complement the information from the nutrition facts label with the list of ingredients to identify toddler foods that are more likely to have added sugars in high proportion. An opportunity exists for nutrition educators and public health professionals to develop educational strategies to heighten this parent population’s skills in determining the nutritional value of these foods and identifying which products are more healthful. In addition, further research is necessary to assess the extent to which parents of toddlers 1) are aware of “how much is too much” sodium and added calories derived from sugar, and 2) are reading labels of food products for small children and whether this impacts point-of-purchase decisions.

At an international level, nutrition transition associated with globalization has predominantly involved increased consumption of processed foods high in sodium, fat and sugar by populations of developing countries (Hawkes, 2006), and the associated increase in the prevalence of obesity, cardiovascular diseases and type 2 diabetes in these countries (Basch, Samuel, & Ethan, 2013). Since preferences for salty and sweet foods established during childhood can have a lifelong influence on dietary habits and health, it is imperative to identify culturally appropriate nutrition education tools for parents to identify toddler foods that are more healthful.
